# Trunk lean mass and its association with 4 different measures of thoracic kyphosis in older community dwelling persons

**DOI:** 10.1371/journal.pone.0174710

**Published:** 2017-04-03

**Authors:** J. Yamamoto, J. Bergstrom, A. Davis, D. Wing, J. T. Schousboe, J. F. Nichols, D. M. Kado

**Affiliations:** 1 University of Hawaii, Honolulu, Hawaii, United States of America; 2 University of California, San Diego, La Jolla, California, United States of America; 3 Park Nicollet Osteoporosis Center and Institute for Research and Education, Minneapolis, Minnesota, United States of America; Garvan Institute of Medical Research, AUSTRALIA

## Abstract

**Background:**

The causes of age-related hyperkyphosis (HK) include osteoporosis, but only 1/3 of those most severely affected have vertebral fractures, suggesting that there are other important, and potentially modifiable causes. We hypothesized that muscle mass and quality may be important determinants of kyphosis in older persons.

**Methods:**

We recruited 72 persons >65 years to participate in a prospective study designed to evaluate kyphosis and fall risk. At the baseline visit, participants had their body composition measures completed using Dual Energy X-ray Absorptiometry (DXA). They had kyphosis measured in either the standing [S] or lying [L] position: 1) Cobb angle from DXA [L]; 2) Debrunner kyphometer [S]; 3) architect’s flexicurve ruler [S]; and 4) blocks method [L]. Multivariable linear/logistic regression analyses were done to assess the association between each body composition and 4 kyphosis measures.

**Results:**

Women (n = 52) were an average age of 76.8 (SD 6.7) and men 80.5 (SD 7.8) years. They reported overall good/excellent health (93%), the average body mass index was 25.3 (SD 4.6) and 35% reported a fall in the past year. Using published cut-offs, about 20–30% were determined to have HK. For the standing assessments of kyphosis only, after adjusting for age, sex, weight and hip BMD, persons with lower TLM were more likely to be hyperkyphotic.

**Conclusions:**

Lower TLM is associated with HK in older persons. The results were stronger when standing measures of kyphosis were used, suggesting that the effects of muscle on thoracic kyphosis are best appreciated under spinal loading conditions.

## Introduction

Hyperkyphosis (HK) is an age-related condition characterized by excess anterior curvature of the thoracic spine. Accentuated kyphosis in older persons has been linked to many adverse health outcomes, including falls, impaired pulmonary function, and earlier mortality [1–4]. While the development of hyperkyphosis is often attributed to osteoporosis and vertebral fractures, almost two-thirds of severe kyphosis cases occur independently of these risk factors [5,6]. Thus, it is likely that other factors such as muscle quality and mass play a significant role in maintaining normal spinal curvature.

Prior research has suggested that paraspinal muscle composition and density are associated with hyperkyphosis in older men and women [7]. In a related study, older non-obese men in the lowest tertile of paraspinal muscle volume (PMV) displayed a greater mean Cobb angle compared to those with high PMV [8]. Back extensor strength (BES) was also found to influence kyphosis, as older women with low BES had greater thoracic kyphosis [9,10]. The results of these studies support the hypothesis that muscle is an important factor in maintaining posture, a concept that may seem obvious, but has never been specifically targeted in a systematic way in terms of helping to better understand the underlying pathophysiology of those suffering from worsening age-related kyphosis progression.

The methods by which spinal muscle was assessed in the above studies are unlikely to have the clinical utility due to expense and radiation exposure from CT scans or specialized equipment needed to measure back extensor strength. As a potential alternative, Dual-energy X-ray absorptiometry (DXA) based body composition measurements provide a cost-effective option compared to CT/MRI, while also minimizing patient exposure to harmful radiation [11]. Body composition including lean mass, fat mass, and bone mineral density (BMD) can be accurately and reliably assessed using DXA, that is already considered the gold standard for osteoporosis screening [12]. Thus, capitalizing on the ability to easily obtain body composition measures during assessments of BMD via DXA, we conducted a cross-sectional study of 72 older persons who had DXA derived body composition data and 4 different measures of kyphosis done during the same visit. We hypothesized that lower trunk lean mass (TLM) would be associated with greater degrees of kyphosis across all 4 measures independent of potential covariates age, sex, weight, and BMD.

## Methods

### Participants

Study participants were community-dwelling ambulatory older men and women recruited from the San Diego area to participate in a prospective study to better understand the association between kyphosis, balance, and risk of falls in persons 65 and older. Participants were recruited from advertisements in the UCSD’s Stein Institute of Research and Aging monthly newsletter, The Stein Institute of Research and Aging website, and through referrals from physicians in the UC San Diego Medical System. All participants were over the age of 65 and English speaking. Participants were excluded from the study if they exhibited advanced disability, end-stage disease, a Mini-mental status exam score <26 (maximum score 30), or severe hearing loss (with or without the use of a hearing aid). The target recruitment goal was 70; due to enrollment enthusiasm and available resources, we enrolled and completed baseline measurements in 72 older adults who comprised this study’s population. The University of California San Diego’s Institutional Review Board approved the study protocol that required written informed consent prior to participation.

### Kyphosis measures

**Fig 1:**
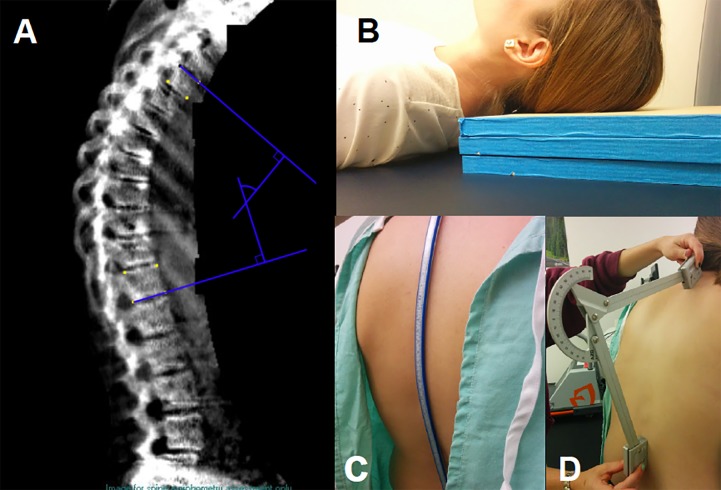
Four measures of kyphosis. A: Cobb angle measurement. B: Blocks method. C: Architect’s flexicurve ruler used to calculate the kyphotic index. D: Debrunner kyphometer.

The degree of kyphosis was measured using 4 methods: Debrunner Kyphometer, Kyphosis Index, Blocks system, and Cobb Angle (Fig 1). The Debrunner Kyphometer is a protractor-like device with parallel arms connected to blocks at each end. The blocks were aligned with the upper (T2) and lower limits (T12) of the thoracic spine allowing the kyphometer to measure the angle of spinal curvature with the patient standing. The Kyphosis Index was calculated from measurements obtained from a flexicurve ruler, a pliable instrument molded to the curvature of the spine from C7 to L5 with the patient in a standing position. The molded-flexicurve was then traced to reflect thoracic and lumbar spinal curvatures. From the tracings, the Kyphosis Index for the thoracic spine was calculated using the formula: (Thoracic Width/Thoracic Length) x 100 [13]. The Blocks system measured kyphosis by stacking 1.7cm thick wooden blocks beneath the participant’s head, while supine, until his/her line of sight was perpendicular to the plane of his/her body. The Cobb Angle was measured from DXA generated lateral spine radiographs taken in the supine position. To calculate the Cobb Angle, lines were drawn from the superior aspect of T4 and the inferior aspect of T12 [14]. The perpendicular intersection of these lines was defined as the Cobb Angle. Inter-rater and intra-rater reliability of all 4 measures raged from 0.63–0.76, with further measurement details previously published [15].

### Bone mineral density and body composition

Total body and regional BMD and body composition were assessed by dual-energy X-ray absorptiometry (DXA) using a Prodigy fan beam densitometer (GE/Lunar, Madison, WI; GE Encore software version 14.1). Bone measurement sites included AP and lateral spine, proximal femur, and total body. Body composition measures of lean and fat mass were determined from total body scans using software-derived standardized regions of interest, which were subsequently confirmed by a DXA technician. Sites of interests included the appendicular region (arms and legs combined), trunk, and whole body. All DXA scans were conducted by the same certified technician. In our laboratory, coefficients of variation (CV) on 30 subjects measured twice with repositioning were 1.05%, 0.8%, 0.85% for the lumbar spine, total hip, and total body BMD, respectively; CVs for total body fat mass and lean mass were 1.46% and 0.55%, respectively.

### Vertebral fracture

Prevalent vertebral fractures using Genant’s semi-quantitative method were assessed by a physician reader (JTS) from vertebral fracture assessments acquired from DXA scans that were performed to obtain bone density and body composition measures [16].

### Questionnaire

Baseline health status (outstanding, excellent, very good, good, or poor), assessment of instrumental activities of daily living, PROMIS measures of self-reported physical function, history of falls in past year, medication use and past medical history were obtained from an interviewer-administered questionnaire.

### Statistical analysis

The means, standard deviations and distributions of each body composition and kyphosis measure were calculated, first examining the entire study population and then by stratifying by sex. All measurements tended to follow a normal distribution, with some sex differences noted in both body composition and kyphosis measures. We used multivariate linear and logistic regression to assess the association of HK with total lean mass, TLM, appendicular lean mass, trunk fat%, trunk fat mass and total fat mass in the entire study sample and in sex-stratified analyses. For the logistic regression analyses, previously defined or study sample derived cut-offs for HK were used: 1) ≥54° for Debrunner; 2) ≥17 for Flexicurve; 3) ≥4 for Blocks; and 4) ≥50° for Cobb Angle [1,17]. In the case of the flexicurve measurement, we could find no previously defined cut-off definition of hyperkyphosis, so we derived the cut-off from the expected prevalence of HK in this population of 20–30%.

To test for potential confounders, we used Pearson correlation coefficients to assess for significant associations between age, sex, weight, height, body mass index (kg/m^2^), prevalent vertebral fracture, and either body composition or kyphosis measures (p < 0.20 used as criterion for entry into multivariable model). Given the sex differences in both body composition and kyphosis measures, we tested for interactions between sex, each body composition and kyphosis measure, but none were significant so only results including both sexes together are presented. Based on the above screening criteria, fully adjusted models included: age, sex, weight, and hip BMD. No adjustments were made for multiple testing due to the “pilot” nature of the study, and its’ intent of hypothesis generation. Data were analyzed using IBM SPSS Statistics (Version 22.0).

## Results

Included in the study were 72 participants, 72% female, ages 65–96 years. Older community-dwelling men (mean age 80.5 ± 7.8 years) and women (mean age 77.8 ± 7.1 years) reported being in good/excellent health (93%), six had prevalent vertebral fractures of grade 2 or more, and 35% of participants reported a fall within the past 12 months (Table 1). Kyphosis measurements were separated according to the patient position during the measurement: standing and supine. The means (SD) of standing kyphotic measures were Flexicurve 13.9 (±4.9) and Debrunner 44.5° (±14) and the supine means were Blocks 3.2 (1.5), and Cobb Angle 42.0°(±12.4) (Table 2).

**Table 1:** Study participant characteristics.

Characteristic	Total N = 72	Men N = 20	Women N = 52
**Age (yr)**	77.8 (7.1)	80.5 (7.8)	76.8 (6.7)
**Self-Report Good or Excellent Health (%)**	67 (93%)	19 (95%)	48 (92%)
**History of falls past year (%)**	25 (35%)	8 (40%)	17 (33%)
**Height (cm)**	164 (10.0)	174 (9.0)*	161 (8.0)
**Weight (kg)**	69.0 (15.0)	79.3 (14.2)*	65.1 (13.4)
**BMI (kg/m**^**2**^**)**	25.3 (4.6)	25.9 (2.8)	25.1 (5.1)
**Total Hip BMD (g/cm**^**2**^**)**	0.89 (0.15)	0.99 (0.18)*	0.85 (0.11)
**Trunk Lean Mass (kg)**	20.2 (4.2)	25.2 (3.8)*	18.4 (2.4)
**Appendicular Lean Mass (kg)**	17.6 (4.3)	22.4 (3.8)*	15.8 (2.9)
**Appendicular Lean Mass/Height (kg/m**^**2**^**)**	6.5 (1.1)	7.3 (0.7)*	6.1 (1.0)

*p < 0.05 for differences between men and women

**Table 2:** Mean kyphosis values and percent prevalence of hyperkyphosis.

Characteristic	Total N = 72	Men N = 20	Women N = 52
**Age (yr)**	77.8 (7.1)	80.5 (7.8)	76.8 (6.7)
**Cobb Angle**	42.0 (12.4)	39.8 (11.4)	42.9 (12.8)
**Cobb Angle ≥ 50 (%)**	21 (30%)	4 (20%)	17 (33%)
**Debrunner**	44.5 (11.8)	47.6 (9.3)	43.2 (12.6)
**Debrunner ≥ 54 (%)**	14 (20%)	4 (20%)	10 (20%)
**Flexicurve**	13.9 (4.9)	14.1 (3.0)	13.8 (5.5)
**Flexicurve ≥ 17 (%)**	18 (25%)	4 (20%)	14 (27%)
**Blocks**	3.2 (1.5)	3.9 (1.6)*	2.9 (1.3)
**Blocks ≥ 4 (%)**	28 (40%)	12 (60%)*	16 (32%)

*p < .05 for differences between men and women

Hyperkyphosis defined as: Cobb Angle ≥50°; Debrunner ≥54°; Flexicurve ≥17; Blocks

Male participants, on average, were more kyphotic than women as measured by Debrunner, flexicurve, and blocks. When setting the criteria for HK ≥ 4 blocks, 60% of males were hyperkyphotic compared to only 32% of women (Table 2). Cobb Angle was the only measure to display a greater degree of kyphosis in women compared to men, though the difference was non-significant (p>.05).

After adjusting for age, sex, weight, and hip BMD, participants with decreased TLM trended towards greater degrees of kyphosis across all 4 measures (p<0.05) (Table 3). Although vertebral fracture did not make screening criteria to be entered into the multivariable model, additional adjustment for prevalent vertebral fracture made no difference in the results. Similarly, using the previously described cut-off threshold(s) to define persons as hyperkyphotic, significant associations were detected between TLM and the standing measures of kyphosis. Participants with less TLM were at greater risk of being hyperkyphotic according to the Debrunner (OR = 1.69, 95% CI: 1.05, 2.70) and Flexicurve (OR = 1.82, 95% CI: 1.18, 2.86) measurements (Table 4). However, using a specific cut-off to define hyperkyphosis with the supine measures, there were no significant associations between TLM and either Blocks or Cobb Angle defined hyperkyphosis.

**Table 3:** Linear regression results of the association of trunk lean mass and kyphosis.

Trunk Lean Mass	Debrunner β (95% CI)	p	Flexicurve β (95% CI)	p	Blocks β (95% CI)	p	Cobb β (95% CI)	p
Unadjusted	-0.17(-0.84; 0.50)	.62	0.11(-0.17; 0.39)	.42	-0.03(-0.11; 0.05)	.47	0.67 (-0.03;1.36)	.06
Adjusted for age & sex	0.15 (-0.84; 1.14)	.77	0.17(-0.23; 0.57)	.40	0.06(-0.04; 0.17)	.24	0.66 (-0.35;1.68)	.20
Adjusted for age, sex, & weight	1.47 (-0.26; 3.20)	.10	0.57(-0.12; 1.26)	.10	0.17(-0.00; 0.35)	.05	1.69 (-0.05;3.43)	.06
Adjusted for age, sex, weight, & hip BMD	**1.93 (0.15; 3.72)**	**.03**	**0.83 (0.14; 1.53)**	**.02**	**0.21(0.03; 0.40)**	**.02**	**2.07 (0.31;3.82)**	**.02**

*Trunk Lean Mass units: per kilogram decrease

**Table 4:** Logistic regression results of the association of trunk lean mass and hyperkyphosis.

Trunk Lean Mass	Debrunner ≥ 54 Odds Ratio	p	Flexicurve ≥ 17 Odds Ratio	p	Blocks ≥ 4 Odds Ratio	p	Cobb ≥ 50 Odds Ratio	p
Unadjusted	1.08 (0.92; 1.27)	.31	1.14 (0.97; 1.32)	.11	0.97 (0.87; 1.09)	.64	1.09 (0.95;1.25)	.21
Adjusted for age & sex	1.12 (0.89; 1.41)	.32	1.16 (0.94; 1.45)	.15	1.12 (0.90; 1.39)	.31	1.03 (0.85; 1.27)	.72
Adjusted for age, sex, & weight	1.41 (0.93; 2.15)	.11	1.56 (1.06; 2.33)	.03	1.13 (0.79; 1.65)	.48	1.30 (0.91; 2.84)	.15
Adjusted for age, sex, weight, & hip BMD	**1.69 (1.05; 2.70)**	**.03**	**1.82 (1.18; 2.86)**	**.01**	1.30 (0.87, 1.96)	.19	1.43 (0.98; 2.08)	.06

*Trunk lean mass units: per kilogram decrease.

With regards to other body composition measures, there were no significant associations detected with any of the four measures of kyphosis, with the exception of one counterintuitive finding: in logistic regression analyses of appendicular lean mass (ALM) and hyperkyphosis measured by the Debrunner kyphometer, those with greater ALM were more likely to be hyperkyphotic (p = 0.03).

## Discussion

We found that TLM is associated with hyperkyphosis in older persons with stronger results observed when measuring kyphosis in the standing position. These findings suggest that the effects of trunk muscles on thoracic kyphosis are best realized under spinal loading conditions. TLM is an essential component in spinal stabilization and a deficit in TLM can affect the spine’s response to weight-bearing stress [17]. Impaired spinal correction can accentuate forward leaning posture, further facilitating kyphotic progression. This association appears limited to lean mass in the truncal region as total body lean mass had no effect on hyperkyphosis. Additional body composition measures such as total fat mass, trunk fat mass, and trunk fat% had no consistent influence on the degree of kyphosis, whether measures in the lying or standing position.

Interestingly, appendicular lean mass displayed a single significant association with hyperkyphosis only when assessed using the Debrunner kyphometer by logistic regression analysis. Given the consistency of findings between TLM across the 4 different measures of kyphosis, whether assessed in linear or logistic regression analyses, we posit that the single counter-intuitive association between greater appendicular mass and Debrunner defined hyperkyphosis most likely represents a false positive finding.

There were some sex differences in the prevalence of hyperkyphosis depending on which measure was used, though with the Cobb angle, Debrunner kyphometer and Flexicurve, the range of 20–33% was consistent with that reported in the literature (see Table 2). The one measure that was notably different between men and women was block-measured kyphosis, with 60% of men and 32% of women defined as hyperkyphotic. Men being twice as kyphotic as women has previously been reported in the Rancho Bernardo Study, although the average number of blocks used to obtain neutral head position was much greater in the current versus earlier study [18]. However, in another cohort, the MrOS study where the blocks measure of kyphosis was also used, men were on average about 7 years older than men in the Rancho Bernardo Study; in MrOS study, those who required ≥4 blocks had a mean age of 80, similar to the mean age of men in the current study [19].

The primary aim of this study was to test the hypothesis that lower trunk lean mass is associated with worse kyphosis, but there are also other health conditions that could account for this finding such as vertebral fractures and degenerative disc disease that might also be associated with low trunk lean mass. Given the close association between vertebral fractures and kyphosis, we considered whether our found association could be explained by underlying prevalent vertebral fractures. Only 8% of our study sample had vertebral fractures and additional adjustment for them did not make any difference in the results.

It is worth noting that with further adjustment for weight and hip bone mineral density, the associations between trunk lean mass and each kyphosis measure were substantially strengthened. Tables 3 and 4 provide the details of how the point estimates and significance changed with additional adjustment for weight and then bone mineral density to the age and sex adjusted models. As might be expected and observed in our study sample, weight was positively correlated with trunk lean mass and negatively correlated with kyphosis so that adjustment for weight led to strengthening of the association. Similarly, bone mineral density was positively correlated with trunk lean mass and negatively correlated with kyphosis so adjustment for bone mineral density further strengthened the point estimate, leading to greater clinical significance.

Currently, there are no standardized clinical recommendations to offer older persons affected by hyperkyphosis. While osteoporosis and hyperkyphosis are certainly related, no currently approved osteoporosis medications have been shown to directly improve or delay the progression of kyphosis. There have been small clinical trials that suggest a benefit of spinal strengthening exercises in improving hyperkyphosis and there are ongoing randomized controlled trials to test whether targeted spinal strengthening exercises may help to improve age-related kyphosis [20,21]. The results of the current study as well as the two previous studies that demonstrated an association between CT measured spinal muscle density and hyperkyphosis suggest that targeted muscle strengthening would be the first and most logical approach to intervention.

Apart from muscle strengthening and postural training through exercise, other treatment modalities are needed to help affected individuals who suffer from progressive age-related kyphosis. With the aging of our population and sarcopenia becoming of increasing interest, one might speculate that investigative drugs such as the myostatin inhibitors or flavonoids may hold promise for improving muscle strength in postural related progressive kyphosis [22,23]. The finding that TLM was more strongly associated with standing measures of kyphosis supports the important role that muscle plays in maintaining optimal posture, particularly during spinal loading.

Our study has notable strengths. To our knowledge, this study was the first to analyze the association of TLM, as measured by DXA, and hyperkyphosis. Although DXA has long been recognized as a vital tool in monitoring osteoporosis and bone health, its capabilities in measuring body composition have often been overlooked. DXA provides an alternative yet effective method to analyze body composition that is cheaper, easier, and safer with minimal radiation exposure. Another study strength was our ability to assess kyphosis across 4 clinically accepted measures, all of which yielded consistent results regardless of patient position (standing or supine).

However, our study also has some limitations. Our sample group, recruited from an urban community, consisted primarily of women (72%) who identified ethnically as Caucasian. This homogeneity may limit the application of our results to the general population. In addition, our participants were all generally in good health as reported by the health survey and reasonably high functioning as evidenced by their ability to transport themselves to/from the clinical laboratory. While the sample group contained several hyperkyphotic individuals, there were no participants with hyperkyphosis severe enough to prevent ambulation. The general good health of our sample may limit the ability of our results to apply to a more frail and/or hyperkyphotic population. In addition, the multiple testing performed in this study increased the probability of detecting false positive findings, but the consistency of results across four different, but related measures of kyphosis, reduces our concern that the reported results were spurious. Lastly, DXA was our sole measure of body composition throughout this study. We were unable to compare our body composition data with CT or MRI for consistency. Furthermore, DXA is very adept at distinguishing lean mass from bone and fat tissue but is unable to delineate specific muscle groups and their position within each region.

In summary, we found that lower TLM is associated with hyperkyphosis in older community dwelling men and women. Our associations were strongest with standing measures of kyphosis suggesting that spinal loading conditions may emphasize deficits in TLM and accentuate thoracic kyphosis. These findings suggest that individuals with lower TLM are at increased risk for hyperkyphosis and its associated morbidities, but given the cross-sectional nature of the study, it is also theoretically possible that the poor posture leads to worse muscle quality or that they are not causally related. Identifying “at-risk” patients earlier through body composition monitoring may be an effective strategy in slowing age-related kyphotic progression. Further research is needed to determine the optimal therapy for individuals with low TLM and whether exercise programs, emerging pharmacologic therapeutics, and/or physical therapy can improve hyperkyphosis and overall quality of life.

## Supporting information

S1 Fig Original source of Fig 1. Four measures of kyphosis as described in Tran et al, “Correlations among four measures of thoracic kyphosis in older adults.” Osteoporos Int 2016; 27:1257.
(PDF)

S1 File Minimal data set. Data used in analyses provided in the attached supplemental file.
(CSV)
