# Supplementary material for: Trunk lean mass and its association with 4 different measures of thoracic kyphosis in older community dwelling persons
Source: PLoS One. 2017 Apr 3;12(4):e0174710. doi: 10.1371/journal.pone.0174710 (PMC5378351; doi:10.1371/journal.pone.0174710)
Supplement: S1 Fig — Four measures of kyphosis as described in Tran et al, “Correlations among four measures of thoracic kyphosis in older adults.” Osteoporos Int 2016; 27:1257. (PDF) [file pone.0174710.s001.pdf]

Correlations among four measures of thoracic kyphosis in older adults

T. H. Tran, D. Wing, A. Davis, J. Bergstrom, J. T. Schousboe, J. F. Nichols & D. M. Kado

Osteoporosis International
With other metabolic bone diseases

ISSN 0937-941X
Volume 27
Number 3

Osteoporos Int (2016) 27:1255-1259
DOI 10.1007/s00198-015-3368-7

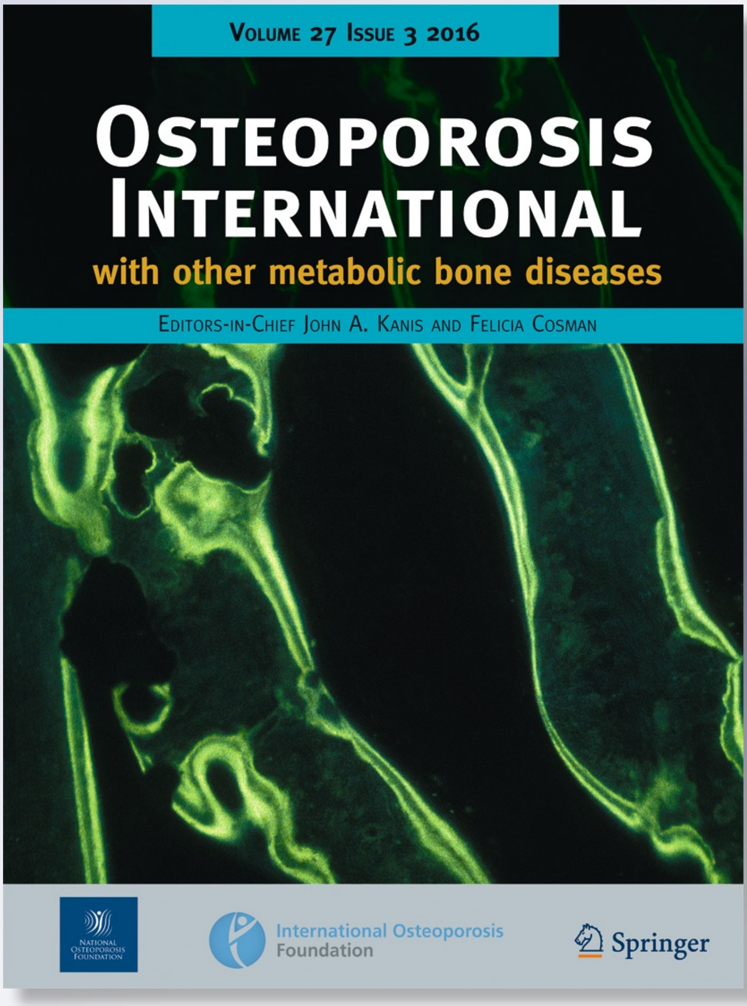

Springer

Your article is protected by copyright and all rights are held exclusively by International Osteoporosis Foundation and National Osteoporosis Foundation. This e-offprint is for personal use only and shall not be self-archived in electronic repositories. If you wish to self-archive your article, please use the accepted manuscript version for posting on your own website. You may further deposit the accepted manuscript version in any repository, provided it is only made publicly available 12 months after official publication or later and provided acknowledgement is given to the original source of publication and a link is inserted to the published article on Springer's website. The link must be accompanied by the following text: "The final publication is available at link.springer.com".

Springer

Author's personal copy

Osteoporos Int (2016) 27:1255–1259
DOI 10.1007/s00198-015-3368-7

SHORT COMMUNICATION

Correlations among four measures of thoracic kyphosis in older adults

T. H. Tran [1,3] · D. Wing [1,3] · A. Davis [1,3] · J. Bergstrom [1,3] · J. T. Schousboe [2,3] · J. F. Nichols [1,3] · D. M. Kado [1,3]

Received: 1 September 2015 / Accepted: 6 October 2015 / Published online: 16 October 2015
© International Osteoporosis Foundation and National Osteoporosis Foundation 2015

Abstract
Summary There are many ways to measure thoracic kyphosis ranging from simple clinical to more complex assessments. We evaluated the correlation among four commonly used kyphosis measures: Cobb angle, Debrunner kyphometer, kyphotic index, and the blocks method. Each measure was correlated with the others, confirming high clinical and research applicability.
Introduction The purpose of this study was to assess the associations among four commonly used measures of thoracic kyphosis in older adults.
Methods Seventy two men and women aged 65–96 were recruited from the San Diego community. Four kyphosis measures were assessed in the same person during a baseline clinic visit. Two measures were done in the lying (L) and two in the standing (ST) position: (1) Cobb angle calculated from dual X-Ray absorptiometry (DXA) images (L), (2) Debrunner kyphometer (DK) angle measured by a protractor (ST), (3) kyphotic index (KI) calculated using an architect's flexicurve ruler (ST), and (4) the blocks method involving counting the number of 1.7 cm-thick blocks required to achieve a neutral head position while lying flat on the DXA table (L). Spearman rank correlation coefficients were used to determine the strength of the association between each kyphosis measure.
Results Using the Cobb angle as the gold standard, the blocks method demonstrated the lowest correlation (r_s=0.63, p<0.0001), the Debrunner method had a moderate correlation (r_s=0.65, p<0.0001), and the kyphotic index had the highest correlation (r_s=0.68, p<0.0001). The correlation was strongest between the kyphotic index and the Debrunner kyphometer (r_s=0.76, p<0.0001).
Conclusion In older men and women, all four measures of thoracic kyphosis were significantly correlated with each other, whether assessed in the lying or standing position. Thus, any of these measures demonstrate both potential clinical and research utility.

Keywords Blocks · Cobb angle · Debrunner kyphometer · Hyperkyphosis · Kyphosis · Kyphotic index

Introduction

Hyperkyphosis is the excessive curvature of the thoracic spine, commonly described as the dowager's hump. Risk factors for this condition include vertebral fractures [1], degenerative disk disease [2], low bone density and bone density loss [3], and spinal extensor muscle weakness [4]. When severe, hyperkyphosis can result in serious health consequences including impaired mobility [5], risk of injurious falls [6], non-spine fractures [7], and earlier mortality [8].

Due to the significant health consequences associated with hyperkyphosis, early diagnosis and treatment may be crucial in reducing complications and associated comorbidities. However, understanding of clinical thresholds at which risk is increased, and before which treatment should be started, is made more challenging because there are multiple ways to assess

✉ D. M. Kado
dkado@ucsd.edu

1 Department of Family Medicine and Public Health, University of California at San Diego, 9500 Gilman Drive #0725, La Jolla, CA 92093-0725, USA

2 Park Nicollet Osteoporosis Center and Institute for Research and Education, Minneapolis, MN, USA

3 Division of Health Policy and Management, University of Minnesota, Minneapolis, MN, USA

Author's personal copy

kyphosis and the degree of correlation between measures has not been well described to date.

Long considered as the gold standard for assessing kyphosis, the Cobb angle has a history dating back to 1948. In short, the Cobb angle is derived from a radiograph of the lateral spine that can be taken in a standing or lying position. The angle is calculated from the interval of the thoracic curve (usually T4 to T12). It has been established as the standard method for quantifying the curvature of the spine due to its reliability and reproducibility, which has been evaluated in scoliosis and kyphosis [9]. However, the Cobb angle is limited by the requirement for a radiograph, which may be cost-ineffective and time consuming when measuring large populations. It also involves radiation exposure that may pose unnecessary health risks, particularly in already at-risk populations. With this in mind, alternative methods to measure kyphosis have been developed. These measures include: (1) the Debrunner kyphometer [10], (2) the kyphotic index, derived from a measurement conducted using a flexicurve ruler [11], and (3) the blocks method [5].

The Debrunner kyphometer and the kyphotic index are both non-invasive measures that are performed with patients in a standing position. The Debrunner kyphometer was designed in 1973 and consists of a protractor attached to two movable arms that when placed at the top and bottom of the thoracic curve, measures the angle of kyphosis. The kyphotic index uses a 60 cm flexicurve ruler, which can be bent in one plane and retain its shape, thus providing a mold of the spinal curve. The mold is traced onto paper and the height of the thoracic spine is determined by drawing a line perpendicular to the apex of the curve. This method can be used to calculate the kyphotic index (width/length × 100). The inter-rater and intra-rater reliability of these two standing measures have been compared in postmenopausal women and showed a strong correlation in the measurement of kyphosis [12]. The validity and reliability have also been investigated between the Cobb angle and the kyphotic index [13], the Cobb angle and the Debrunner kyphometer [14], and between all the three aforementioned measures [15].

The blocks method assesses a patient's degree of kyphosis by using 1.7 cm-thick blocks placed under the participant's head while lying supine. Hyperkyphotic individuals, when lying supine, are unable to lie in a neutral position without hyperextension of the neck. Blocks are placed under the head until a neutral position is achieved, with a greater number of blocks being indicative of a greater severity of kyphosis. It has been used to assess the degree of kyphosis in older adults [5], but its validity has not yet been compared to the Cobb angle, Debrunner kyphometer, or kyphotic index methods of measurement.

The use of a fast, non-radiologic, non-invasive method for assessing kyphosis offers benefits for clinical and research practices. To our knowledge, no study has systematically used and reported results of correlation between all the four major measures of kyphosis done concurrently. Thus, the purpose of this study was to determine correlations between the kyphotic index, the Debrunner kyphometer, the blocks methods of measurement, and the gold standard radiographically derived Cobb angle.

Methods

Subjects

72 persons aged 65–96 were recruited from the San Diego community. Kyphosis measures were conducted at the Exercise and Physical Activity Resource Center (EPARC) at UC San Diego as part of a larger assessment battery to investigate how degree of kyphosis is associated with balance and future falls. After providing informed consent, participants disclosed their medical history and underwent a comprehensive health assessment that measured their physical function, balance, bone mineral density, and body composition. This study was approved by the Institutional Review Board of the University of California, San Diego (UCSD).

Measurement of kyphosis

Four measures of kyphosis were gathered from the same person, two done in the lying (L) and two in the standing (ST) position (see Fig. 1):

- The *Cobb angle* is calculated from a dual-energy X-ray absorptiometry (DXA) radiograph of the lateral thoracic spine, obtained in the supine position on a GE Lunar densitometer with a rotating C-arm, using the dual-energy mode. Lines were drawn from the upper border of the T4 vertebral body and the lower border of the T12 vertebral body, from which perpendicular lines were erected. The angle of intersection of these perpendicular lines comprises the Cobb angle. When unable to clearly the T4 vertebral body, T5 was used as the upper end instead. The edges of the vertebral bodies used to calculate the Cobb angle were identified by a physician rheumatologist considered an expert in the field of spinal measurement (JTS). (L)
- The *Debrunner Kyphometer* (DK) angle is measured by a moveable protractor. The arms of the device were positioned at bony landmarks at the top and bottom of the thoracic curve to measure the angle of kyphosis. The upper arm was positioned between the interspace of T2–T3, while the lower arm was positioned on the interspace between T11–T12. [10]. (ST)
- The *Kyphotic Index* (KI) is calculated using an architect's flexicurve ruler, which was molded onto the spinous

Author's personal copy

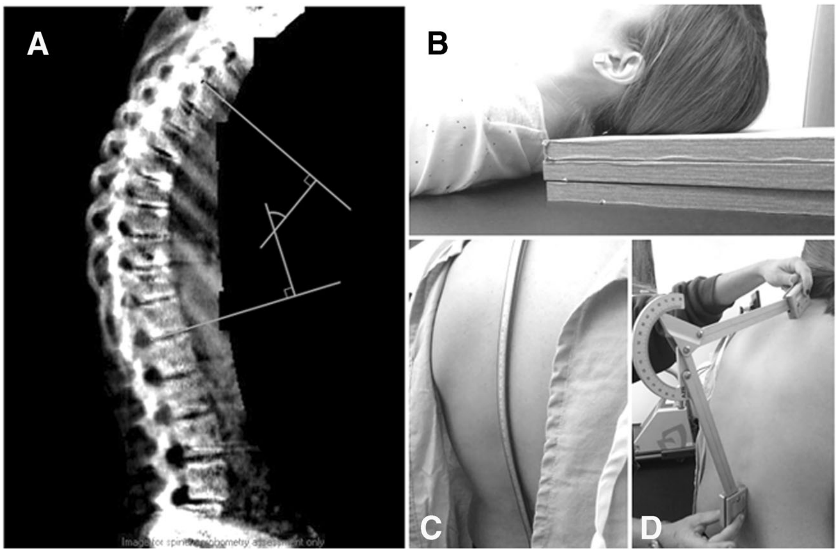

Fig. 1 Four measures of hyperkyphosis. **a** Cobb angle measurement. **b** Blocks method. **c** Architect's flexicurve ruler used to calculate the kyphotic index. **d** Debrunner kyphometer

process of C7 to the interspace of L5–S1. This mold was traced onto paper, and a vertical line was drawn to align the C7 spinous process and the interspace of L5–S1. A horizontal line was drawn connecting the apex of the thoracic curve to the vertical line. The kyphotic index was calculated as a function of the thoracic length and thoracic width using standardized procedures previously described (width/length × 100). [11]. (ST)

- The *Blocks method* involves counting the number of 1.7-cm thick blocks required to achieve a neutral head position with the subject lying in a supine position on the DXA table. This was the final measure conducted in the sequence during the participant's clinic visit. (L)

Statistical analysis

Spearman rank correlations coefficients were used to determine the strength of the association between each kyphosis measure. The kyphotic index and Debrunner methods of measurements were conducted by two or more raters per participant in order to evaluate inter-rater reliability. For the Cobb angle, inter-rater reliability was evaluated in a random sample of 20 films with the readers blinded to each other's results. To evaluate intra-rater reliability, 20 films were read twice after a 4 week interval. Since all kyphosis measures were normally distributed with the exception of the blocks kyphosis measurement that is an ordinal variable (range 0–7), Pearson correlation analyses were also performed. As similar results were obtained, the Spearman rank correlation coefficient data are reported. All analyses were performed using SPSS, version 22 (IBM Corporation, Armonk, NY).

Results

The characteristics of the 52 women and 20 men in the study are reported in Table 1. Their ages ranged from 65 to 96 and the mean age was 76.9 (±6.7). The mean BMI was 25.4 (±4.5) kg/m^2, and the mean percent body fat was 36.2 % (±7.2 %). Using a hyperkyphosis cutoff of a Cobb angle exceeding 50°, 21 of the 72 participants (29.2 %) were classified as hyperkyphotic [7]. Similarly, using previous published literature to guide thresholds to define hyperkyphosis, 14 (20 %) of those with the Debrunner (≥54), 18 (25 %) of those with the kyphotic index (≥17), and 28 (40 %) of those with the blocks (≥4 blocks) were diagnosed as hyperkyphotic [1, 8, 16, 17].

Of the 72 participants, 70 measurements were gathered for the blocks method, 72 measurements for the kyphotic index, and 71 measurements for the Cobb angle and Debrunner methods. Correlations were only investigated in measurements with values that were available for both of the respective methods. The Cobb angles were assessed using the T5-T12 interval in radiographs of 16 (23 %) participants, and T4-T12 in the radiographs of the remaining 55 participants. Tests of inter-rater reliability were excellent to outstanding for the following kyphosis measurement methods: (1) kyphotic index, ICC = 0.933; (2) Debrunner kyphometer, ICC = 0.993; and (3) Cobb angle were ICC = 0.968. A single rater conducted all block measures of kyphosis, but other studies have published inter-rater reliabilities of ≥0.85 [17].

Using the Cobb angle as the gold standard, the blocks method demonstrated the lowest correlation ($r_s = 0.63$, $p < 0.0001$), the Debrunner method had a moderate correlation ($r_s = 0.65$, $p < 0.0001$), and the kyphotic index had the highest correlation ($r_s = 0.68$, $p < 0.0001$).

Author's personal copy

Table 2 Correlations between measures of kyphosis. Spearman rank correlation coefficients (r_s) were used to determine the strength of association between two lying (L) and two standing (S) measures of kyphosis: the Cobb angle (L), blocks method (L), kyphotic index (S), and Debrunner kyphometer (S). All correlations are significant (p<0.0001)

	Cobb Angle (L)	Block (L)	Kyphotic Index (S)	Debrunner (S)
Cobb Angle (L)	1.000	0.632	0.684	0.652
Block (L)	0.632	1.000	0.680	0.744
Kyphotic Index (S)	0.684	0.680	1.000	0.764
Debrunner (S)	0.652	0.744	0.764	1.000

The correlation was strongest between the Debrunner kyphometer and the kyphotic index (r_s= 0.76, p< 0.0001). A full description of the correlations between measures is presented in Table 2.

Discussion

In older men and women, all four measures of kyphosis were significantly correlated with each other, whether assessed in the lying or standing position. As such, all measures can be used with a reasonable degree of reliability to measure degree(s) of kyphosis.

As expected, the correlation was strongest between the two standing measures, the flexicurve and Debrunner kyphometer with an r_s of 0.76. Differences in correlation between the standing and lying measures may be attributed to the participants' active maintenance of posture while in the upright position. Specifically, standing measures require the activation of muscles of the thoracic and cervical spine that can affect the measured degree of kyphosis [21]. Spinal loading forces not activated while supine, but engaged while upright, should serve to accentuate the degree of kyphosis, particularly if spinal muscle strength is compromised. Several studies suggest that back extensor strength or spinal muscle volume/density affects the degree of kyphosis in older persons [4, 18–20].

For measures done in the lying position, the correlation between the blocks method and Cobb angle was the weakest (r_s=0.63). While this result may seem counterintuitive, the comparatively low correlation may be due to at least two reasons. First, the blocks method is a relatively crude and subjective measure that is an incremental/non-continuous variable with a range from 0 to ≥7 whereas the Cobb angle is a measure of degrees with a much wider range of possible values. Second, the blocks measure captures aspects of the cervical spine not measured by the Cobb angle, which marks as its endpoints the superior edge of T4 or T5 and the inferior edge of T12. Thus, the blocks measure captures an overall sense of the cervical and upper thoracic spinal configuration and cannot differentiate between thoracic and cervical kyphosis, whereas the Cobb angle specifically measures thoracic kyphosis. Third, we did not test the inter-rater reliability of the blocks method; lower precision of this measure could weaken its association with other measures of kyphosis.

A strength of this study was that all four measures were conducted in succession at the baseline visit. Inter-rater reliability of all measures also showed strong correlations. A limitation of this study was that only self-sufficient patients who were capable of traveling to the measurement site were included. Given health problems associated with kyphosis, participants with severe cases of hyperkyphosis may have been under-represented. A second limitation was that the four measures were conducted in a predetermined order that could

Table 1 Cohort characteristics. Kyphosis was assessed using four measures in 72 elderly persons in the San Diego community. Each of the measures were conducted in sequence during the baseline visit

Characteristic	Females (N=52)	Males (N=20)	Total (N=72)		
	Mean (SD)	Mean (SD)	Mean (SD)	Median	Range
Age (year)	76.8 (6.7)	80.5 (7.8)	77.8 (7.1)	77.0	65.0–96.0
Height (cm)	160.9 (8.3)	174.2 (9.3)	164.6 (10.4)	163.8	133.4–189.2
Weight (kg)	65.1 (13.4)	79.3 (14.2)	69.0 (15.0)	70.1	40.9–108.5
BMI (kg/m2)	25.1 (5.1)	25.9 (2.8)	25.3 (4.6)	25.3	17.9–36.2
% Body Fat	38.5 (6.8)	30.4 (5.0)	36.2 (7.3)	36.1	19.3–51.8
Cobb Angle	42.9 (12.8)	39.8 (11.4)	42.0 (12.4)	42.0	16.0–66.5
Debrunner	43.2 (12.6)	47.6 (9.3)	44.5 (11.8)	46.0	17.5–74.5
Kyphotic Index	13.8 (5.5)	14.1 (3.0)	13.9 (4.9)	12.8	2.0–34.5
Block (# of blocks)	2.9 (1.3)	3.9 (1.6)	3.2 (1.5)	3.0	1.0–7.0

Author's personal copy

have lead to unintentional measurement bias. However, the Cobb angle and flexicurve index values of kyphosis were calculated months after the raw data were collected, and the raters were unaware of any of the other kyphosis results at the time, making measurement bias unlikely. Third, the traditional Cobb angle of kyphosis is measured from standing lateral spine radiographs where our measure was done from a vertebral fracture assessment DXA measure obtained in the lying position. However, other large epidemiological studies that have measured the Cobb angle from films obtained in a lying position have demonstrated that the Cobb angle ascertained in the lying position is associated with poor health outcomes, indicating measurement validity [7]. It has not been previously investigated whether or not measures of kyphosis obtained in the standing position correlate better with adverse outcomes than in the lying position. Importantly, these data are not applicable to vertebral fracture assessment images obtained in the lateral decubitus position, since the validity and accuracy of Cobb angle measurements on such images has not been established.

In conclusion, the correlations between the four measures of kyphosis examined in the present study indicate that when radiographs are not available, the Debrunner kyphometer, the flexicurve derived kyphotic index, and the blocks method are all sufficient and comparatively cost-effective measures to determine severity of kyphosis in clinical and/or research settings.

Acknowledgments This work was supported by the University of California, San Diego's Department of Family Medicine and Public Health, the University of California Academic Senate the UCSD Stein Institute for Research on Aging, and NIH/NIAMS AR06828.

Compliance with ethical standards

Conflict of interest None.

References

1. Ensrud KE, Black DM, Harris F et al (1997) Correlates of kyphosis in older women. The Fracture Intervention Trial Research Group. J Am Geriatr Soc 45(6):682–687
2. Schneider DL, von Mühlen D, Barrett-Connor E et al (2004) Kyphosis does not equal vertebral fractures: the Rancho Bernardo study. J Rheumatol 31(4):747–752
3. Kado DM, Huang MH, Karlamangla AS et al (2013) Factors associated with kyphosis progression in older women: 15 years' experience in the study of osteoporotic fractures. J Bone Miner Res 28(1):179–187. doi:10.1002/jbmr.1728
4. Sinaki M, Itoi E, Roger JW et al (1996) Correlation of back extensor strength with thoracic kyphosis and lumbar lordosis in estrogen-deficient women. Am J Phys Med Rehabil 75(5):370–374
5. Kado DM, Huang MH, Barrett-Connor E et al (2005) Hyperkyphotic posture and poor physical functional ability in older community-dwelling men and women: the Rancho Bernardo study. J Gerontol A Biol Sci Med Sci 60(5):633–637
6. Kado DM, Huang MH, Nguyen CB et al (2007) Hyperkyphotic posture and risk of injurious falls in older persons: the Rancho Bernardo Study. J Gerontol A Biol Sci Med Sci 62(6):652–657
7. Kado DM, Miller-Martinez D, Lui LY (2014) Hyperkyphosis, kyphosis progression, and risk of non-spine fractures in older community dwelling women: the study of osteoporotic fractures (SOF). J Bone Miner Res 29(10):2210–2216. doi:10.1002/jbmr.2251
8. Kado DM, Lui LY, Ensrud KE et al (2009) Hyperkyphosis predicts mortality independent of vertebral osteoporosis in older women. Ann Intern Med 150(10):681–687
9. Carman DL, Browne RH, Birch JG (2009) Measurement of scoliosis and kyphosis radiographs. Intraobserver and interobserver variation. J Bone Joint Surg Am 72(3):328–333
10. Öhlén G, Spangfort E, Tingvall C (1989) Measurement of spinal sagittal configuration and mobility with Debrunner's kyphometer. Spine 14:580–583
11. Milne JS, Lauder IJ (1976) The relationship of kyphosis to the shape of vertebral bodies. Ann Hum Biol 3(2):173–179
12. Lundon KM, Li AM, Bibershtein S (1998) Interrater and intrarater reliability in the measurement of kyphosis in postmenopausal women with osteoporosis. Spine 23(18):1978–1985
13. Azadinia F, Kamyab M, Behtash H et al (2014) The validity and reliability of noninvasive methods for measuring kyphosis. J Spinal Disord Tech 27(6):E212–E218. doi:10.1097/BSD.0b013e31829a3574
14. Kado DM, Christianson L, Palermo L et al (2006) Comparing a supine radiologic versus standing clinical measurement of kyphosis in older women: the fracture intervention trial. Spine 31(4):463–467
15. Greendale GA, Nili NS, Huang MH et al (2011) The reliability and validity of three non-radiological measures of thoracic kyphosis and their relations to the standing radiological Cobb angle. Osteoporos Int 22(6):1897–1905. doi:10.1007/s00198-010-1422-z
16. Ettinger B, Black DM, Palermo L et al (1994) Kyphosis in older women and its relation to back pain, disability, and osteopenia: the study of osteoporotic fractures. Osteoporos Int 4(1):55–60
17. Katzman WB, Harrison SL, Fink HA et al (2015) Physical function in older men with hyperkyphosis. J Gerontol A Biol Sci Med Sci 70(5):635–640. doi:10.1093/gerona/glu213
18. Katzman WB, Vittinghoff E, Ensrud K et al (2011) Increasing kyphosis predicts worsening mobility in older community-dwelling women: a prospective cohort study. J Am Geriatr Soc 59(1):96–100. doi:10.1111/j.1532-5415.2010.03214.x
19. Katzman WB, Miller-Martinez D, Marshall LM et al (2014) Kyphosis and paraspinal muscle composition in older men: a cross-sectional study for the Osteoporotic Fractures in Men (MrOS) research group. BMC Musculoskelet Disord 15:19. doi:10.1186/1471-2474-15-19
20. Yamamoto J, Bergstrom J, Davis A, Wing D, Nichols J, Kado D (2015) Trunk lean mass and its association with 3 measures of kyphosis in older dwelling persons. J Am Geriatr Soc 63(Suppl 1):S14
